# Qualitative Assessment of Soil Carbon in a Rehabilitated Forest Using Fourier Transform Infrared Spectroscopy

**DOI:** 10.1100/tsw.2011.54

**Published:** 2011-03-07

**Authors:** Huck-Ywih Ch'ng, Osumanu Haruna Ahmed, Nik Muhamad Ab. Majid

**Affiliations:** ^1^ Department of Crop Science, Faculty of Agriculture and Food Sciences, Universiti Putra Malaysia Bintulu Sarawak Campus, Bintulu, Sarawak, Malaysia; ^2^ Department of Forest Management, Faculty of Forestry, Universiti Putra Malaysia, Serdang, Selangor, Malaysia

**Keywords:** soil carbon, carbon buildup, FTIR, carbon in humic acids, rehabilitated forest

## Abstract

Logging and poor shifting cultivation negatively affect initial soil carbon (C) storage, especially at the initial stage of deforestation, as these practices lead to global warming. As a result, an afforestation program is needed to mitigate this problem. This study assessed initial soil C buildup of rehabilitated forests using Fourier transform infrared (FTIR) spectroscopy. The relatively high E4/E6 values of humic acids (HAs) in the rehabilitated forest indicate prominence of aliphatic components, suggesting that the HAs were of low molecular weight. The total acidity, carboxylic (-COOH) and phenolic (-OH) of the rehabilitated forest were found to be consistent with the ranges reported by other researchers. The spectra of all locations were similar because there was no significant difference in the quantities of C in humic acids (CHA) regardless of forest age and soil depth. The spectra showed distinct absorbance at 3290, 1720, 1630, 1510, 1460, 1380, and 1270 cm^-1^. Increase of band at 1630 and 1510 cm^-1^ from 0–20 to 40–60 cm were observed, suggesting C buildup from the lowest depths 20–40 and 40–60 cm. However, the CHA content in the soil depths was not different. The band at 1630 cm^-1^ was assigned to carboxylic and aromatic groups. Increase in peak intensity at 1510 cm^-1^ was because C/N ratio increased with increasing soil depth. This indicates that decomposition rate decreased with increasing soil depth and decreased with CHA. The finding suggests that FTIR spectroscopy enables the assessment of C composition functional group buildup at different depths and ages.

## INTRODUCTION

Currently, land-use changes are becoming the largest contribution of anthropogenic emissions besides fossil fuel emissions[1]. This is because land-use changes affect carbon (C) storage and also the amount of biomass and C stored in vegetation, thus causing a decrease in soil fertility[2]. Land use in Malaysia, especially Sarawak, has changed significantly because of transmigration and changes in the rural economy. Excessive logging, mining, and oil palm cultivation (shifting cultivation) contribute to deforestation in Sarawak, Malaysia[3]. According to Lasco[4], deforested areas covered with grasses and annual crops have C densities <40 mg $ha^{-1}$, which is much less than natural forests. Lasco[4] also found that conversion of natural forests to tree plantations and perennial crops reduced C density by at least 50% compared to natural forests. A study by Davidson and Ackerman[5] also found that conversion of forest land to agricultural land decreased C stock by 20–50%.

The Intergovernmental Panel on Climate Change (IPCC) stated in its first scientific assessment in 1990 that human activities contributing to greenhouse gases from land-use changes could lead to an unknown consequence of mankind[6]. As a result of concerns about climate change, land use, land-use change, and forestry (LULUCF) have been recognized as playing the role of C source and sink in relation to change in land cover and carbon stocks[7]. Processes that lead to soil organic carbon (SOC) sequestration are conversion of biomass into humus, including humic acids (HAs), aggregation to prevent C oxidation, and translocation of C into subsoil[8]. Forests ecosystems cover approximately 4.1 billion ha globally[9]. Forest soils play an important role in the global C cycle[10]. Understanding the mechanism and factors contributing to C buildup in soil is important to enhance natural sinks of C sequestration to mitigate the climate change[11].

According to West and Post[12], land use and management, as well as climate and soil texture, influence not only soil organic matter (SOM), total C, and stable C content (derived from HA extracted from the soil), but also its composition of functional groups, e.g., carboxylic and phenolic. The advances in instrumentation and techniques allow structural determination and chemical composition of organic substances[13]. Fourier transform infrared (FTIR) spectroscopy has been used for the characterization of complex organic macromolecules, such as humic substances, and qualitatively and nondestructively the principal classes of chemical groups of which SOM is comprised[13].

Montagnini and Porras[14] stated that facilitating reforestation can be an effective C management approach. To date, many studies have been done to estimate regional C storage and national C budgets of temperate forests in many countries. However, there is a lack of qualitative information on the soil C buildup in the initial stage of rehabilitated forests in tropical areas such as Malaysia. Since CHA realistically reflects soil C storage[8,15], it is essential to use FTIR to assess qualitatively the C buildup in afforestation programs that contribute to C storage.

The objective of this work was to assess the soil C buildup in the initial stages of a rehabilitated forest at different soil depths using FTIR spectroscopy.

## MATERIALS AND METHODS

This study was conducted in a rehabilitated forest of the University Putra Malaysia, Bintulu Sarawak Campus area (latitude 03°12 N and longitude 113°02 E), with mean annual rainfall, temperature, and relative humidity of 2933 mm, 27°C, and 80%, respectively. The area was previously abandoned after shifting cultivation and it has been rehabilitated since 1991 by planting indigenous timber species from the family Dipterocarpaceae and Non-Dipterocarpaceae, such as Anacardiacea, Annonaceae, Bombacaceae, Compositae, and Meliaceae. The soil texture of the rehabilitated forest is sandy loam and was typical of Nyalau Series (*Typic Tualemkuts*). An auger was used to collect soil samples from the 1- to 7-year-old rehabilitated forest areas. Soil sampling for all of the ages of the rehabilitated forest was done at the same time to avoid bias. The size of each experimental plot was 30 × 40 m. A total of 10 soil samples were taken at random depths of 0–20, 20–40, and 40–60 cm in each plot for each age. Each sample was a bulk of three subsamples.

The soil samples were air dried, pounded, and sieved to pass a 2-mm size. Soil pH was determined in a 1:2.5 ratio of soil:distilled water suspension and/or 1 M KCl using glass electrode[16]. The soil total N was determined using the micro-Kjeldahl method[17]. The soil total C and CHA were analyzed using a CHNS analyzer[18].

The extraction of HA was done using the method described by Stevenson[19], but with some modifications. A 10-g soil sample was placed in a polyethylene centrifuge bottle, after which 100 mL of 0.1 M NaOH solution was added and the bottle was tightly closed with a rubber stopper[20]. The samples were equilibrated at room temperature on a reciprocal mechanical shaker at 180 rpm for 12 h. After the extraction period, the walls of the bottle were washed using distilled water and the mixture was centrifuged at 16,211 *g* for 15 min. The dark-colored supernatant liquors containing the HA were decanted, filtered through glass-wool, and the pH of the solution was adjusted to 1.0 with 6 M HCl. The HA was equilibrated at room temperature for 12 h. After 12 h, the supernatant (fulvic acids) was siphoned off from the acidified extract. The remainder of the suspension was transferred to polyethylene bottles and the HA was centrifuged. A purification process was conducted to purify the HA. It was done by using the method described by Ahmed et al.[21] with some modifications. The HA was purified by suspension in 100 mL of distilled water and centrifuged at 16,211 *g* for 10 min, and the supernatant was decanted. The washed HA was oven dried at 40$^{o}$C to a constant weight. The yield of the HA was expressed as percentage of the weight of soil used. The humification level of HA was determined using $E_4/E_6$ Perkin Elmer Lambda 25 UV/VIS spectroscopy[19,22]. The carboxylic (-COOH), phenolic (-OH) functional groups and total acidity were determined by the method described by Inbar et al.[23].

The HAs extracted were analyzed for the C functional groups spectrum using FTIR. Two milligrams of the HA samples were pressed with (FTIR grade) KBr (1:100) to a pellet. The pellet was immediately measured in a spectrometer after preparation under ambient conditions using the transmission mode. The measurement was carried out in the mid-IR range from 4000 to 400 cm$^{-1}$ with a Perkin-Elmer 2000 FTIR spectrometer. The resolution was set to 4 cm$^{-1}$, 32 scans were recorded, averaged for each spectrum, and corrected against ambient air as background. Corrected peak heights were used for FTIR analysis and were obtained using Perkin-Elmer software Spectrum for Windows 1.3 (1996). In order to quantify the relative changes in the FTIR spectra and for the spectra comparison, relative absorbance (rA) was calculated by dividing the corrected peak height of a distinct peak by the sum of the heights of all peaks at 3290, 1720, 1630, 1510, 1450, 1380, and 1270 cm$^{-1}$, and multiplied by 100 (rA = % of the sum of all peak heights from 3290 to 1270 cm$^{-1}$).

Data obtained from the laboratory analysis were analyzed using the Statistical Analysis System (SAS) Version 9.2. One-way analysis of variance (ANOVA) was used to detect significant differences between pH (water) and pH (KCl), soil total C, CHA, total N, C/N ratio, and HA at different soil depths and different ages of the rehabilitated forest. Tukey's HSD test ($p \leq 0.05$) was used to separate the means of the bulk densities, SOM, soil total C, CHA, total N, C/N ratio, and HA between soil depths and different ages of the rehabilitated forest.

## RESULTS AND DISCUSSION

### Soil Samples Analysis

The pH (Table 1) of the different ages of the rehabilitated forest, regardless of soil depths, was typical of Ultisols. There was significant difference between the pH (1 M KCl) of the rehabilitated forest soils at 0–20, 20–40, and 40–60 cm, except for the 1-year-old rehabilitated forest soil. The pH (1 M KCl) at 0–20 cm of the 2-, 3-, 4-, 5-, 6-, and 7-year-old rehabilitated forest soils were lower than those at 20–40 and 40–60 cm, except for the 1-year-old rehabilitated forest soil, which showed no significant difference in pH (1 M KCl) in the three different depths. On the other hand, there was no significant difference between the pH (water) of the rehabilitated forest soil at 0–20, 20–40, and 40–60 cm except for the 7-year-old rehabilitated forest soil. The higher pH (1 M KCl) values of the soil at 20–40 and 40–60 cm compared to those at 0–20 cm of the 2-, 3-, 4-, and 7-year-old soils could be due to the leaching of basic cations from 0–20 cm to 20–40 and 40–60 cm. The significant difference between soil pH of the different ages of the rehabilitated forest regardless of soil depth suggests that different ages of the rehabilitated forest at different locations had a significant effect on the soil pH.

**TABLE 1**
**pH at Different Soil Depths and Comparison of pH of Different Ages of a Rehabilitated Forest**

| Rehabilitated Forest | pH (Water) | pH (1 M KCl) |
|---|---|---|
| (a) 1-year-old | | |
| 0–20 cm | $4.70^{aAB}$ | $3.99^{aA}$ |
| 20–40 cm | $4.72^{aAB}$ | $4.02^{aA}$ |
| 40–60 cm | $4.73^{aAB}$ | $4.03^{aA}$ |
| (b) 2-year-old | | |
| 0–20 cm | $4.61^{aBC}$ | $3.73^{aC}$ |
| 20–40 cm | $4.62^{aAB}$ | $3.79^{bB}$ |
| 40–60 cm | $4.67^{aBC}$ | $3.81^{bB}$ |
| (c) 3-year-old | | |
| 0–20 cm | $4.66^{aAB}$ | $3.68^{aCD}$ |
| 20–40 cm | $4.69^{aAB}$ | $3.71^{bC}$ |
| 40–60 cm | $4.70^{aAB}$ | $3.72^{bC}$ |
| (d) 4-year-old | | |
| 0–20 cm | $4.84^{aA}$ | $3.76^{aC}$ |
| 20–40 cm | $4.88^{aA}$ | $3.80^{bB}$ |
| 40–60 cm | $4.90^{aA}$ | $3.84^{bB}$ |
| (e) 5-year-old | | |
| 0–20 cm | $4.34^{aC}$ | $3.84^{aB}$ |
| 20–40 cm | $4.42^{aC}$ | $3.85^{aB}$ |
| 40–60 cm | $4.44^{aC}$ | $3.87^{aB}$ |
| (f) 6-year-old | | |
| 0–20 cm | $4.61^{aBC}$ | $3.09^{aE}$ |
| 20–40 cm | $4.63^{aB}$ | $3.09^{aD}$ |
| 40–60 cm | $4.63^{aBC}$ | $3.10^{aD}$ |
| (g) 7-year-old | | |
| 0–20 cm | $4.44^{aC}$ | $3.61^{aD}$ |
| 20–40 cm | $4.56^{bBC}$ | $3.69^{bC}$ |
| 40–60 cm | $4.60^{bBC}$ | $3.73^{bC}$ |

The soil total N content (Table 2) obtained for the different ages of the rehabilitated forest was in the range reported by Lee et al.[15]. The percentage of soil total N of the rehabilitated forests generally decreased down the soil profile. However, there was no significant difference between the soil total N content in the 2-year-old rehabilitated forest as well as between the different ages of the rehabilitated forest within the same depth. The significant decrease in percentage of soil total N of the rehabilitated forest down the soil profile and this observation was consistent with the general observation that soil N decreases with increasing soil depth because of a decrease in organic N. The soil total N regardless of soil depth and age of the rehabilitated forest were typical of Ultisols. The absence of a significant difference of total N between the topsoil and subsoil could be attributed to the soil texture, which was sandy loam, whereby there was a possibility of the N being leached out from 0–20 cm and accumulated in the 20–40 cm.

**TABLE 2**
**Soil Total N and C/N Ratio at Different Soil Depths and Comparison of Soil Total N and C/N Ratio of Different Ages of a Rehabilitated Forest**

| Rehabilitated Forest | Total N (%) | C/N Ratio |
|---|---|---|
| (a) 1-year-old | | |
| 0–20 cm | $0.102^{aA}$ | $7.574^{aBC}$ |
| 20–40 cm | $0.056^{bA}$ | $10.321^{bB}$ |
| 40–60 cm | $0.056^{bA}$ | $12.918^{cAB}$ |
| (b) 2-year-old | | |
| 0–20 cm | $0.093^{aA}$ | $8.741^{aB}$ |
| 20–40 cm | $0.084^{aA}$ | $11.630^{bAB}$ |
| 40–60 cm | $0.074^{aA}$ | $14.138^{cA}$ |
| (c) 3-year-old | | |
| 0–20 cm | $0.102^{aA}$ | $10.912^{aA}$ |
| 20–40 cm | $0.065^{bA}$ | $11.243^{abAB}$ |
| 40–60 cm | $0.056^{bA}$ | $14.384^{bA}$ |
| (d) 4-year-old | | |
| 0–20 cm | $0.112^{aA}$ | $9.924^{aA}$ |
| 20–40 cm | $0.074^{abA}$ | $12.936^{bA}$ |
| 40–60 cm | $0.065^{bA}$ | $13.154^{bAB}$ |
| (e) 5-year-old | | |
| 0–20 cm | $0.084^{aA}$ | $7.113^{aC}$ |
| 20–40 cm | $0.056^{abA}$ | $7.456^{aC}$ |
| 40–60 cm | $0.028^{bB}$ | $10.600^{abC}$ |
| (f) 6-year-old | | |
| 0–20 cm | $0.084^{aA}$ | $8.347^{aAB}$ |
| 20–40 cm | $0.065^{abA}$ | $9.708^{abC}$ |
| 40–60 cm | $0.046^{bA}$ | $11.628^{bBC}$ |
| (g) 7-year-old | | |
| 0–20 cm | $0.112^{aA}$ | $9.320^{aAB}$ |
| 20–40 cm | $0.065^{abA}$ | $11.720^{abB}$ |
| 40–60 cm | $0.056^{bA}$ | $12.006^{bB}$ |

The C/N ratio significantly increased down the profile for the different ages of the rehabilitated forest (Table 2). This suggests that there was more humification at 0–20 cm than at 20–40 and 40–60 cm[24,25]. The significant difference observed in the C/N ratios of the different ages of the rehabilitated forest at 0–20, 20–40, and 40–60 cm suggest differences in the humification levels because it is impossible to obtain a uniform humification degree across the different ages of the forest and down the soil profiles. The lower C/N ratios of the 1-, 2-, and 5-year-old rehabilitated forest soils compared to those of the 3-, 6-, and 7-year-old forest soils could be due to the significant accumulation of N at a depth of 0–20 cm as discussed previously.

There was a significant difference in the percentages of soil total C of the different ages of the rehabilitated forest with increasing soil depth (Table 3). The quantities of total C in the different ages of the rehabilitated forest in the topsoil were significantly different from those of subsoil except for the 4-year-old rehabilitated forest soil. The lower content of C in the subsoil was due to the lack of N for efficient conversion of biomass C to humus C, which is required for humification of biomass returned to soil. It also suggests that higher decomposition of biomass may have occurred through the decomposition of plant and animal residues, root exudates, and living and dead microorganisms. In addition, pH has association with total C. The acidic nature of the soil may have limited the abundance of microbes and their ability to decompose biomass to release more C[26].

**TABLE 3**
**Soil Total C and Corresponding Quantities (mg ha$^{-1}$) at Different Soil Depths and Comparison between Different Ages of a Rehabilitated Forest**

| Rehabilitated Forest | Soil Total C (%) | Quantity of Total C (mg ha$^{-1}$) |
|---|---|---|
| (a) 1-year-old | | |
| 0–20 cm | $0.764^{aBC}$ | $18.736^{aB}$ |
| 20–40 cm | $0.529^{abBC}$ | $18.559^{aAB}$ |
| 40–60 cm | $0.473^{bBC}$ | $14.720^{bAB}$ |
| (b) 2-year-old | | |
| 0–20 cm | $1.297^{aA}$ | $30.804^{aA}$ |
| 20–40 cm | $0.837^{abAB}$ | $24.850^{abA}$ |
| 40–60 cm | $0.611^{bABC}$ | $19.373^{bA}$ |
| (c) 3-year-old | | |
| 0–20 cm | $1.134^{aAB}$ | $21.848^{aAB}$ |
| 20–40 cm | $0.687^{bAB}$ | $17.701^{bAB}$ |
| 40–60 cm | $0.668^{bAB}$ | $18.760^{bAB}$ |
| (d) 4-year-old | | |
| 0–20 cm | $1.065^{aAB}$ | $17.384^{aB}$ |
| 20–40 cm | $0.947^{abA}$ | $18.441^{aAB}$ |
| 40–60 cm | $0.815^{bA}$ | $17.868^{aAB}$ |
| (e) 5-year-old | | |
| 0–20 cm | $0.578^{aC}$ | $14.729^{aB}$ |
| 20–40 cm | $0.326^{bC}$ | $10.580^{bB}$ |
| 40–60 cm | $0.296^{bC}$ | $11.054^{bB}$ |
| (f) 6-year-old | | |
| 0–20 cm | $0.702^{aBC}$ | $17.632^{aB}$ |
| 20–40 cm | $0.611^{abABC}$ | $16.656^{abAB}$ |
| 40–60 cm | $0.488^{bABC}$ | $13.632^{bB}$ |
| (g) 7-year-old | | |
| 0–20 cm | $1.288^{aA}$ | $24.639^{aAB}$ |
| 20–40 cm | $0.587^{bBC}$ | $13.901^{bB}$ |
| 40–60 cm | $0.537^{bABC}$ | $17.086^{bAB}$ |

The percentages of HA yields and corresponding quantities in mg ha$^{-1}$ of the rehabilitated forest at the three different depths were statistically different (Table 4). The percentages and corresponding quantities of HA significantly decreased down the soil profile, except for the 1-year-old rehabilitated forest soil, whereby the percentage and corresponding yield at 20–40 cm was lower than at 40–60 cm. The HA yields also showed significant differences between different ages of the rehabilitated forest. This suggests that there was more humification at 0–20 cm than at 20–40 and 40–60 cm[27]. In addition, the lack of sufficient N for efficient conversion of biomass C to humus C, which is required for humification of biomass returned to soil, was also one of the reasons behind this observation.

**TABLE 4**
**HA Yields and Corresponding Quantities (mg ha$^{-1}$) at Different Soil Depths and Comparison between Different Ages of a Rehabilitated Forest**

| Rehabilitated Forest | HA Yield (%) | Quantity of HA (mg ha$^{-1}$) |
|---|---|---|
| (a) 1-year-old | | |
| 0–20 cm | $0.284^{aAB}$ | $6.963^{aAB}$ |
| 20–40 cm | $0.047^{bB}$ | $1.461^{bB}$ |
| 40–60 cm | $0.298^{aA}$ | $9.294^{aA}$ |
| (b) 2-year-old | | |
| 0–20 cm | $0.540^{aA}$ | $13.032^{aA}$ |
| 20–40 cm | $0.196^{bAB}$ | $5.822^{bAB}$ |
| 40–60 cm | $0.062^{cB}$ | $1.973^{bC}$ |
| (c) 3-year-old | | |
| 0–20 cm | $0.327^{aAB}$ | $6.304^{aAB}$ |
| 20–40 cm | $0.059^{bB}$ | $1.492^{bB}$ |
| 40–60 cm | $0.016^{bBC}$ | $0.455^{bC}$ |
| (d) 4-year-old | | |
| 0–20 cm | $0.386^{aAB}$ | $6.305^{aAB}$ |
| 20–40 cm | $0.320^{abA}$ | $6.240^{abA}$ |
| 40–60 cm | $0.213^{bA}$ | $4.669^{bB}$ |
| (e) 5-year-old | | |
| 0–20 cm | $0.228^{aAB}$ | $5.807^{aAB}$ |
| 20–40 cm | $0.180^{abAB}$ | $5.842^{aAB}$ |
| 40–60 cm | $0.089^{bB}$ | $3.023^{Bb}$ |
| (f) 6-year-old | | |
| 0–20 cm | $0.132^{aB}$ | $3.315^{Ab}$ |
| 20–40 cm | $0.049^{bB}$ | $1.351^{bB}$ |
| 40–60 cm | $0.008^{bC}$ | $0.240^{bC}$ |
| (g) 7-year-old | | |
| 0–20 cm | $0.322^{aAB}$ | $6.162^{aAB}$ |
| 20–40 cm | $0.050^{bB}$ | $1.233^{bB}$ |
| 40–60 cm | $0.057^{bBC}$ | $1.826^{bC}$ |

The relatively high $E_4/E_6$ values (Table 5) in the rehabilitated forest indicate prominence of aliphatic components or the HA in this study were of low molecular weights[19,28]. The effectiveness of washing the HA with distilled water is to indicate its purity without altering its chemical characteristics. The total acidity, carboxylic (-COOH) and phenolic (-OH) of the rehabilitated forest were found to be consistent with the ranges reported by other researchers[29,30].

**TABLE 5**
**Comparison of Ranges of Phenolic, Carboxylic, Total Acidity, and $E_4/E_6$ Ratio of HA of Different Ages of a Rehabilitated Forest**

| Rehabilitated Forest | E4/E6 | Range | Carboxylic | Range | Phenolic | Range | Total Acidity | Range |
|---|---|---|---|---|---|---|---|---|
| | | | cmol kg-1 | | | | | |
| (a) 1-year-old | | | | | | | | |
| 0–20 cm | 7.850 | 6–8* | 500 | 240–540* | 400 | 150–440** | 900 | 390–980* |
| 20–40 cm | 8.144 | | 550 | | 400 | | 950 | |
| 40–60 cm | 7.715 | | 500 | | 400 | | 900 | |
| (b) 2-year-old | | | | | | | | |
| 0–20 cm | 6.628 | | 510 | | 360 | | 870 | |
| 20–40 cm | 7.150 | | 450 | | 360 | | 810 | |
| 40–60 cm | 6.995 | | 500 | | 360 | | 860 | |
| (c) 3-year-old | | | | | | | | |
| 0–20 cm | 7.052 | | 530 | | 420 | | 950 | |
| 20–40 cm | 6.995 | | 550 | | 380 | | 930 | |
| 40–60 cm | 7.150 | | 500 | | 400 | | 900 | |
| (d) 4-year-old | | | | | | | | |
| 0–20 cm | 7.509 | | 530 | | 400 | | 930 | |
| 20–40 cm | 7.588 | | 560 | | 400 | | 960 | |
| 40–60 cm | 7.052 | | 560 | | 400 | | 960 | |
| (e) 5-year-old | | | | | | | | |
| 0–20 cm | 7.621 | | 600 | | 400 | | 1000 | |
| 20–40 cm | 7.154 | | 490 | | 400 | | 890 | |
| 40–60 cm | 7.150 | | 530 | | 400 | | 930 | |
| (f) 6-year-old | | | | | | | | |
| 0–20 cm | 7.621 | | 600 | | 400 | | 1000 | |
| 20–40 cm | 7.154 | | 490 | | 400 | | 890 | |
| 40–60 cm | 7.509 | | 530 | | 400 | | 930 | |
| (g) 7-year-old | | | | | | | | |
| 0–20 cm | 7.150 | | 550 | | 400 | | 870 | |
| 20–40 cm | 7.052 | | 510 | | 400 | | 910 | |
| 40–60 cm | 7.154 | | 530 | | 400 | | 930 | |

* Tan[30].

** Schnitzer and Preston[29].

There was no significant difference in the quantity of CHA for the different ages of the rehabilitated forest at soil depths of 0–20, 20–40, and 40–60 cm (Table 6). The quantities of CHA from the 1- to 7-year-old rehabilitated forest soils at the depth of 0–20 cm were generally higher than those at 20–40 and 40–60 cm, although there was no significant difference between the depths. This shows that planting the exotic species of trees in the rehabilitated forest at initial stages (until 7 years old) does not exert any difference in the amount of C sequestered in the Ultisols. Since the CHA is more stable, it is more realistic to quantify the amount of C sequestered in setting up the rehabilitated forest at initial stages[27].

**TABLE 6**
**Soil Stable C and Corresponding Quantities (mg ha$^{-1}$) at Different Soil Depths and Comparison between Different Ages of a Rehabilitated Forest**

| Rehabilitated Forest | Soil Stable C (%) | Quantity of Stable C (mg ha$^{-1}$) |
|---|---|---|
| (a) 1-year-old | | |
| 0–20 cm | $37.267^{aA}$ | $2.594^{aA}$ |
| 20–40 cm | $37.177^{aA}$ | $0.543^{bA}$ |
| 40–60 cm | $35.940^{aA}$ | $3.340^{aA}$ |
| (b) 2-year-old | | |
| 0–20 cm | $43.169^{aA}$ | $5.625^{aA}$ |
| 20–40 cm | $34.678^{aA}$ | $2.019^{abA}$ |
| 40–60 cm | $41.924^{aA}$ | $0.827^{bA}$ |
| (c) 3-year-old | | |
| 0–20 cm | $34.272^{aA}$ | $2.160^{aA}$ |
| 20–40 cm | $34.256^{aA}$ | $0.511^{bA}$ |
| 40–60 cm | $29.337^{aA}$ | $0.133^{bA}$ |
| (d) 4-year-old | | |
| 0–20 cm | $39.556^{aA}$ | $2.494^{aA}$ |
| 20–40 cm | $36.777^{aA}$ | $2.294^{aA}$ |
| 40–60 cm | $39.134^{aA}$ | $1.827^{aA}$ |
| (e) 5-year-old | | |
| 0–20 cm | $34.973^{aA}$ | $2.030^{aA}$ |
| 20–40 cm | $35.916^{aA}$ | $2.098^{aA}$ |
| 40–60 cm | $35.889^{aA}$ | $1.084^{bA}$ |
| (f) 6-year-old | | |
| 0–20 cm | $43.644^{aA}$ | $1.446^{aA}$ |
| 20–40 cm | $33.432^{bA}$ | $0.452^{bA}$ |
| 40–60 cm | $38.641^{abA}$ | $0.093^{bA}$ |
| (g) 7-year-old | | |
| 0–20 cm | $31.919^{bA}$ | $1.966^{aA}$ |
| 20–40 cm | $43.290^{aA}$ | $0.533^{bA}$ |
| 40–60 cm | $31.891^{bA}$ | $0.582^{bA}$ |

## FTIR Spectra

The spectra characteristics of C buildup in the rehabilitated forest soils are shown in Figs. 1–4. In general, the spectra characteristics of all the locations were similar and showed similar peak patterns. The spectra generally showed distinct absorbance at 3290, 1720, 1630, 1510, 1460, 1380, and 1270 $cm^{-1}$ (Table 7). This could be due to the fact that there was no significant difference in the quantities of stable C between different ages of the rehabilitated forest and at the three different soil depths.

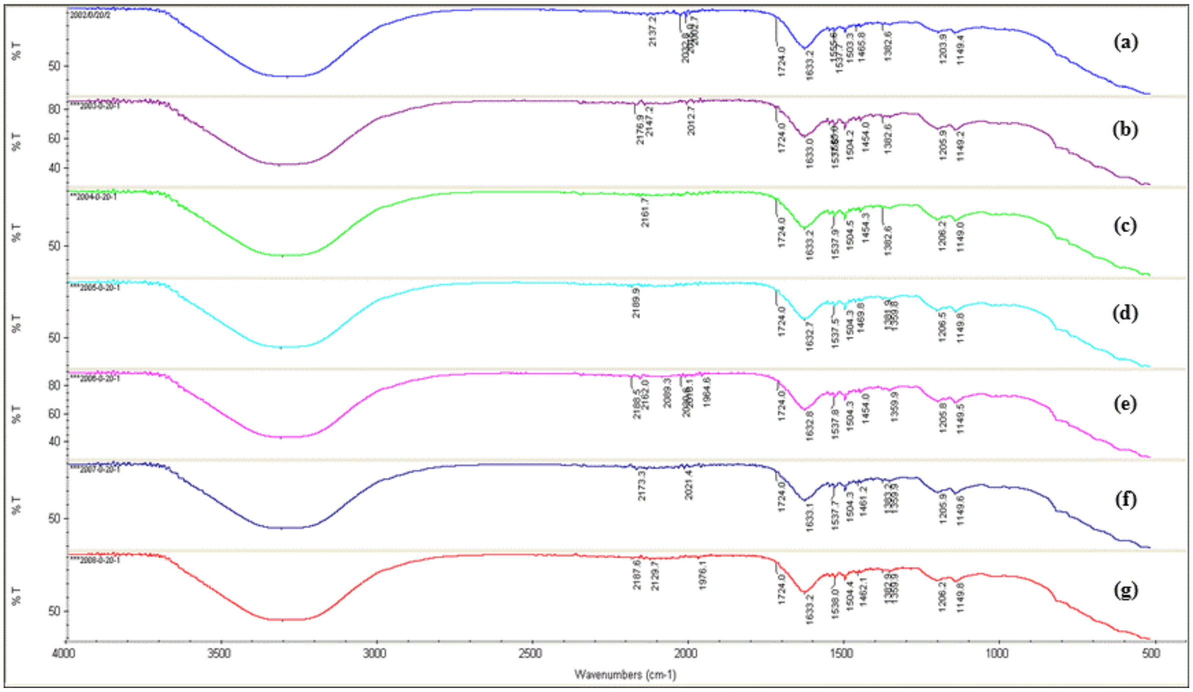

**FIGURE 1.** Comparison of FTIR spectra of C buildup in the different ages of the rehabilitated forest at 0–20 cm. Alphabet letters in brackets denote ages of the rehabilitated forest; (a) 1-year-old rehabilitated forest; (b) 2-year-old rehabilitated forest; (c) 3-year-old rehabilitated forest; (d) 4-year-old rehabilitated forest; (e) 5-year-old rehabilitated forest; (f) 6-year-old rehabilitated forest; (g) 7-year-old rehabilitated forest.

The bands at 3920 $cm^{-1}$ (Table 7) represent stretching of O-H and N-H[25]. The carboxylic band at 1720 $cm^{-1}$ forms only a small shoulder on the main band at about 1630 $cm^{-1}$. The distinct band 1630 $cm^{-1}$ was assigned to C=O vibrations of carboxylates and aromatic vibrations[2,19]. The bands at 1510, 1460, 1380, and 1270 $cm^{-1}$ were attributed to CH and NH (amide II) bending motion, molecule skeleton vibration, and carbon oxygen bond vibration[13]. Bands in this region are highly mixed, representing coupled vibrations, and the analyzed samples are chemically highly complex, containing a number of substances. Thus, it is not possible to assign them to one specific functional group or to a specific substance[19]. The peak at 1510 $cm^{-1}$ was due to the amide II vibrations and to aromatic C=C vibrations. However, the intensity of these bands increased from soil depths of 20–40 to 40–60 cm[19]. The increase in band was in line with the increase of C/N ratio with increasing soil depth and it reflects that decomposition rate slowed down as the soil depth increased. The relative absorbance of the band 3920 $cm^{-1}$ (O-H and N-H stretching) was found to be different within different ages of the rehabilitated forest. The relative absorbance band at 1630 $cm^{-1}$ slightly increased from the topsoil to the subsoil. This suggests that there was an increase in aromatic and carboxylic groups during litter degradation[28].

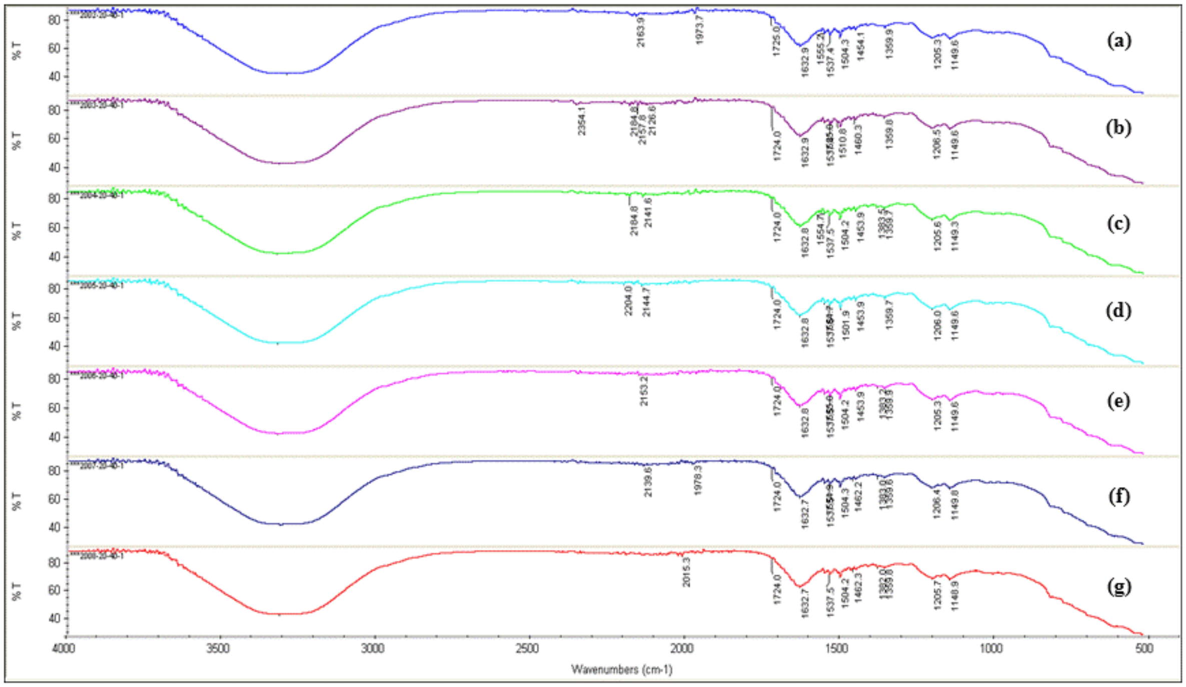

**FIGURE 2.** Comparison of FTIR spectra of C buildup in the different ages of the rehabilitated forest at 20–40 cm. Alphabet letters in brackets denote ages of the rehabilitated forest; (a) 1-year-old rehabilitated forest; (b) 2-year-old rehabilitated forest; (c) 3-year-old rehabilitated forest; (d) 4-year-old rehabilitated forest; (e) 5-year-old rehabilitated forest; (f) 6-year-old rehabilitated forest; (g) 7-year-old rehabilitated forest.

The 1630/3920 and 1630/1510 ratios increased from 20–40 to 40–60 cm (Fig. 4). This represents an increase in carboxylates and aromaticity, and a decomposition of structural properties of the forest litter substances that are responsible for the band 1510 cm$^{-1}$. They also reflect a decrease of aliphatic chains during decomposition of forest organic litter[19].

## CONCLUSION

The relatively high $E_4/E_6$ values of HAs indicate prominence of aliphatic components and the HAs were of low molecular weight. The total acidity, carboxylic (-COOH) and phenolic (-OH) of the rehabilitated forest were found to be consistent with the ranges reported by other researchers. The spectra of C buildup in the rehabilitated forest soils were similar and showed similar peak patterns. The spectra generally showed distinct absorbance at 3290, 1720, 1630, 1510, 1460, 1380, and 1270 cm$^{-1}$. There were no significant differences in the CHA of the different ages of the rehabilitated forest. This finding basically leads to the production of similar spectra and peak patterns. Increase of the bands at 1630 and 1510 cm$^{-1}$ from 0–20 to 40–60 cm were observed and suggests that C was observed to build up from the lowest depths 20–40 and 40–60 cm. However, the CHA content in the three different soil depths were not statistically different. The band at 1630 cm$^{-1}$ was assigned to carboxylic and aromatic groups. The increase in the peak intensity at 1510 cm$^{-1}$ was in line with the increase of C/N ratio with increasing soil depth, and reflects that decomposition rate slowed down as the soil depth increased and decreased with the CHA. The intensity peaks of 1510–1270 cm$^{-1}$ in analysis of FTIR absorbance serve as an identification and evaluation of decomposition rate in the rehabilitated forest soils. It can be used for identification and distinction of soil depths. This finding suggests that FTIR spectroscopy enables the assessment of C composition functional group buildup at different depths and ages.

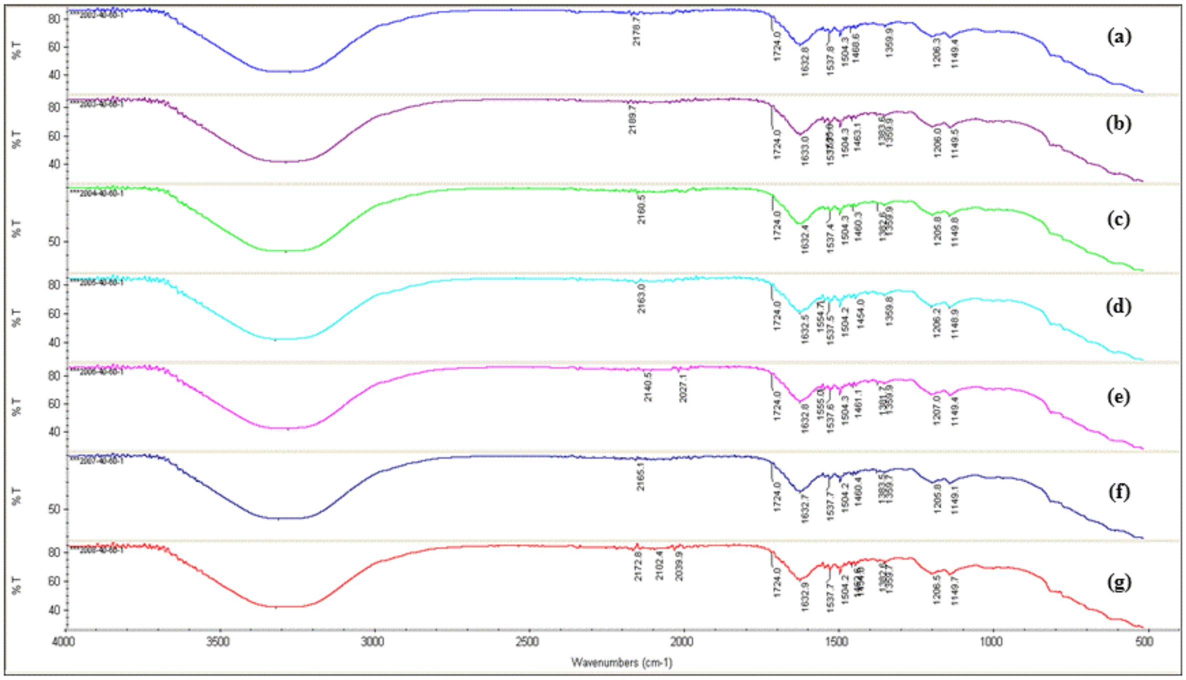

**FIGURE 3.** Comparison of FTIR spectra of C buildup in the different ages of the rehabilitated forest at 40–60 cm. Alphabet letters in brackets denote ages of the rehabilitated forest; (a) 1-year-old rehabilitated forest; (b) 2-year-old rehabilitated forest; (c) 3-year-old rehabilitated forest; (d) 4-year-old rehabilitated forest; (e) 5-year-old rehabilitated forest; (f) 6-year-old rehabilitated forest; (g) 7-year-old rehabilitated forest.

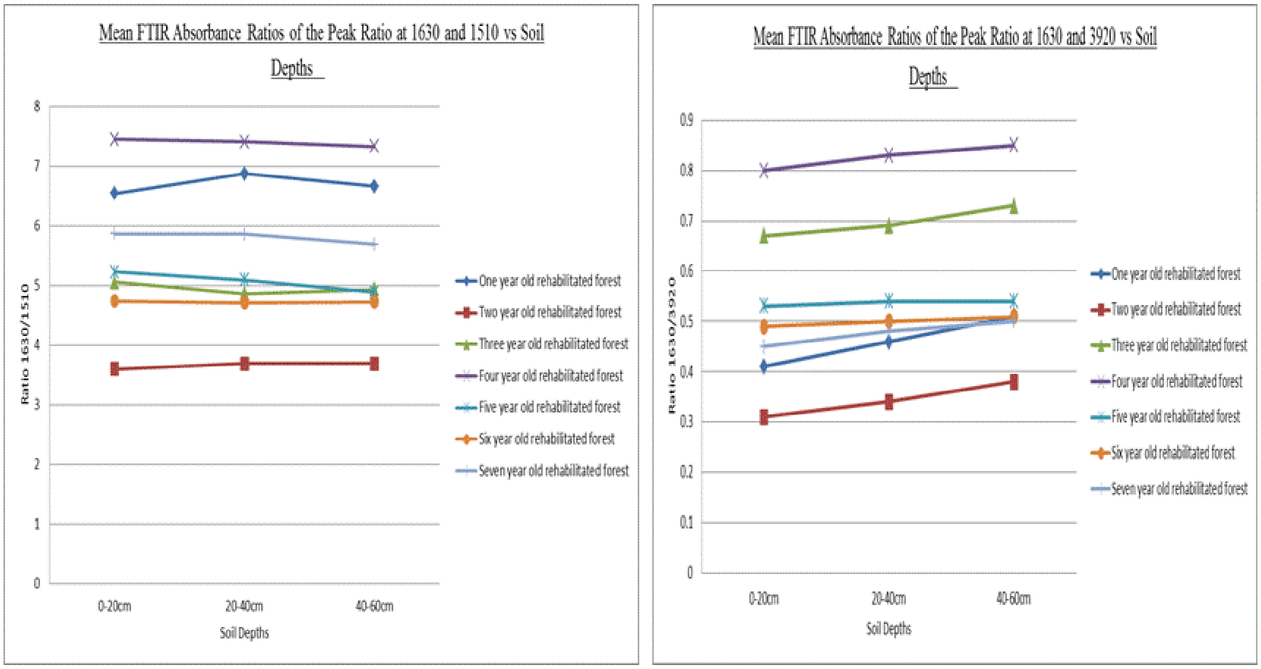

**FIGURE 4.** FTIR absorbance ratios of peak ratio at 1630 and 1510 $cm^{-1}$ and 1630 and 3920 $cm^{-1}$ and soil depths.

**TABLE 7**
**Relative Absorbance of the Sum of All Selected Peak Heights of the FTIR Spectra of Different Soil Depths and Ages of a Rehabilitated Forest**

| Rehabilitated Forest | Relative Absorbance (%) | | | | | | |
|---|---|---|---|---|---|---|---|
| | 3290 | 1720 | 1630 | 1510 | 1460 | 1380 | 1270 |
| | $cm^{-1}$ | | | | | | |
| 1-year-old | | | | | | | |
| 0–20 cm | 62.62 | 0.63 | 25.62 | 3.92 | 1.63 | 1.66 | 3.89 |
| 20–40 cm | 61.18 | 0.82 | 28.25 | 4.11 | 1.22 | 0.97 | 3.45 |
| 40–60 cm | 59.32 | 0.89 | 30.10 | 4.52 | 1.04 | 0.87 | 3.22 |
| 2-year-old | | | | | | | |
| 0–20 cm | 62.96 | 1.39 | 19.55 | 5.43 | 3.10 | 1.99 | 5.58 |
| 20–40 cm | 62.31 | 1.54 | 21.34 | 5.78 | 2.94 | 0.97 | 5.12 |
| 40–60 cm | 60.36 | 1.68 | 23.12 | 6.26 | 2.76 | 0.86 | 4.96 |
| 3-year-old | | | | | | | |
| 0–20 cm | 48.69 | 0.51 | 32.56 | 6.43 | 4.73 | 1.24 | 5.82 |
| 20–40 cm | 48.61 | 0.52 | 33.43 | 6.87 | 4.03 | 1.11 | 5.43 |
| 40–60 cm | 47.57 | 0.59 | 34.76 | 7.04 | 3.89 | 1.03 | 5.12 |
| 4-year-old | | | | | | | |
| 0–20 cm | 44.01 | 1.57 | 35.17 | 4.72 | 3.44 | 1.72 | 9.36 |
| 20–40 cm | 43.59 | 1.62 | 36.22 | 4.89 | 3.01 | 1.57 | 9.10 |
| 40–60 cm | 43.41 | 1.71 | 36.98 | 5.04 | 2.89 | 1.04 | 8.93 |
| 5-year-old | | | | | | | |
| 0–20 cm | 53.57 | 0.78 | 28.32 | 5.41 | 2.74 | 3.16 | 6.02 |
| 20–40 cm | 53.80 | 0.83 | 28.87 | 5.67 | 2.54 | 2.42 | 5.87 |
| 40–60 cm | 53.73 | 0.98 | 29.19 | 5.96 | 2.28 | 2.21 | 5.65 |
| 6-year-old | | | | | | | |
| 0–20 cm | 57.26 | 1.02 | 27.81 | 5.87 | 0.77 | 1.39 | 5.92 |
| 20–40 cm | 57.06 | 1.21 | 28.25 | 6.00 | 0.69 | 1.01 | 5.78 |
| 40–60 cm | 56.68 | 1.44 | 28.98 | 6.13 | 0.51 | 0.66 | 5.60 |
| 7-year-old | | | | | | | |
| 0–20 cm | 60.34 | 0.86 | 27.43 | 4.67 | 0.56 | 1.35 | 4.79 |
| 20–40 cm | 59.75 | 1.01 | 28.65 | 4.89 | 0.40 | 0.72 | 4.58 |
| 40–60 cm | 58.77 | 1.22 | 29.48 | 5.18 | 0.31 | 0.63 | 4.41 |

## ACKNOWLEDGMENTS

The authors acknowledge the UPM-Mitsubishi Corporation Forest Rehabilitation Research Project and Universiti Putra Malaysia, Bintulu Sarawak Campus.
